# Loss of Lean Mass in Rheumatoid Arthritis Is Associated With Loss of Total and Visceral Fat

**DOI:** 10.1002/jcsm.70229

**Published:** 2026-02-11

**Authors:** Joshua F. Baker, Jon T. Giles, Babette Zemel, Katherine D. Wysham, Jin Long, Mary Leonard, Patricia Katz

**Affiliations:** ^1^ Corporal Michael J. Crescenz VA Medical Center Philadelphia Pennsylvania USA; ^2^ University of Pennsylvania School of Medicine Philadelphia Pennsylvania USA; ^3^ Department of Epidemiology and Biostatistics University of Pennsylvania Philadelphia Pennsylvania USA; ^4^ Cedars‐Sinai Medical Center Los Angeles California USA; ^5^ Children's Hospital of Philadelphia Philadelphia Pennsylvania USA; ^6^ VA Puget Sound Health Care System Seattle Washington USA; ^7^ Stanford University Palo Alto California USA; ^8^ University of California San Francisco San Francisco California USA

**Keywords:** adiposity, rheumatoid arthritis, sarcopenia

## Abstract

**Purpose:**

Rheumatoid cachexia has been described as a process of concurrent muscle loss and gain of adipose tissue. We evaluated longitudinal changes in body composition in patients with rheumatoid arthritis (RA) to evaluate the changes in adiposity that accompany loss of lean mass.

**Methods:**

We combined and assessed three independent longitudinal RA cohorts that included assessments of body composition. Whole body DXA was performed in all participants to quantify appendicular lean mass index (ALMI, kg/m^2^) and fat mass index (FMI, kg/m^2^). Independent associations between loss of ALMI during follow‐up and FMI over the same time‐period were assessed adjusting for age, sex, race, baseline body composition and study using mixed‐effects regression to account for clustering by study. Changes in adipokines (adiponectin and leptin) were also assessed over time in similar models. Visceral fat area was determined from DXA (cm^2^) in one of the cohorts and was also assessed.

**Results:**

Among 451 patients with a mean (SD) age of 58.3 (10.5), the mean (SD) ALMI was 6.97 (1.41) kg/m^2^. Longitudinal analyses were conducted in 361 participants with follow‐up data [average follow‐up 2.65 (0.71) years]. Of these, 195 lost lean mass (experienced a negative change in ALMI during follow‐up), while 166 gained lean mass. Participants that lost lean mass had greater reductions in BMI [−0.77 (95% CI: −1.21, −0.33) v. +1.07 (95% CI: 0.56, 1.59)], greater reductions in FMI [−0.17 (95% CI: −0.48, 0.14) v. +0.46 (95% CI: 0.08, 0.83) *p* = 0.07] and a greater odds of having a reduction in FMI [OR: 2.30 (1.31, 4.05) *p* = 0.004], and had declining leptin levels and visceral fat area. Associations were strongest among those with high FMI at baseline.

**Conclusions:**

In RA, loss of lean mass tends to occur in the context of a loss of weight and a loss of both total and visceral adiposity. These observations help to inform our understanding of the mechanisms leading to loss of muscle and rheumatoid cachexia in RA as well as to inform potential screening practices.

## 1 Introduction

Rheumatoid cachexia has often been described as a condition‐associated loss of muscle with stable or even gains in fat mass [1]. This description stems from studies that have identified both greater adiposity and lower lean mass among patients with rheumatoid arthritis (RA) compared to controls [1, 2, 3, 4, 5, 6]. These cross‐sectional observations have led to an assumption that both processes occur simultaneously, though this has not been directly studied in longitudinal datasets to our knowledge.

Prior work has also shown that deficits in total lean mass and muscle density in RA compared to controls are observed primarily among those with lower fat mass [7]. This observation does not support the hypothesis that loss of lean mass occurs simultaneously with gains in adiposity. Gains in fat mass among patients with RA might occur outside the setting of cachexia with potential causes including the effects of treatments such as prednisone, low physical activity due to loss of function or gains in adiposity as part of normal aging. Thus, while patients with RA are at risk of obesity, increased visceral adiposity, and sarcopenia, these outcomes may occur through distinct processes and have different risk factors. With the exception of bariatric surgery or other interventions associated with significant weight loss [8], few studies in any population have defined relationships between lean mass and fat mass over time in a population at risk of cachexia.

To address this question, we studied a large cohort of patients with body composition assessments performed over time in order to determine if loss of lean mass over several years is accompanied by changes in fat mass. We hypothesized that patients who lost muscle over time would also be more likely to lose fat over the same period of time.

## 2 Methods

### 2.1 Study Sample

We combined and assessed three independent cohorts of patients with RA as previously described. In each cohort, body composition assessments were performed at baseline and at a follow‐up visit approximately 2–4 years later. The internal review board at each institution approved each respective study and all subjects gave written informed consent. We performed a complete case analysis, excluding participants that did not have a follow‐up measure to allow determination of change from baseline in key exposures and outcomes.

1. University of California San Francisco (UCSF) Cohort (*N* = 141): Details regarding this study cohort have been previously published [9, 10]. The majority of research participants were drawn from the UCSF RA Panel Study between 2007 and 2009. RA Panel participants who lived in the greater San Francisco area were recruited for in‐person assessments, including measurement of body composition. Exclusion criteria were non–English speaking, age < 18 years, current daily oral prednisone dose > 50 mg, current pregnancy, uncorrected vision problems that interfered with reading and patients who had undergone joint replacement within 1 year.
2. University of Pennsylvania (Penn) Cohort (*N* = 113): The Penn cohort was initiated in 2012. Subjects in the Penn cohort were recruited from the University of Pennsylvania Rheumatology practices and the Philadelphia Veterans Affairs Medical Center and consisted of individuals aged 18–75 years who met 2010 American College of Rheumatology criteria for RA. Subjects with juvenile idiopathic arthritis (or another inflammatory arthritis), active cancer, a history of chronic diseases known to affect bone health (e.g., chronic kidney disease, liver disease and malabsorption syndromes) or pregnancy were excluded. One subject was excluded because her weight exceeded the limit for the DXA machine (300 pounds).
3. Evaluation of Subclinical Cardiovascular Disease and Predictors of Events in RA Study (ESCAPE RA) Cohort (*N* = 190): The ESCAPE RA cohort study recruited men and women between October 2004 and May 2006 [11]. Briefly, patients with RA followed at the Johns Hopkins Arthritis Center or referred from local rheumatologists were enrolled, all of whom met American College of Rheumatology 1987 classification criteria for RA, were 45–84 years of age and did not report any prior pre‐specified cardiovascular events or procedures. Subjects weighing > 300 pounds were excluded due to weight limitations of the imaging equipment.

### 2.2 Whole‐Body DXA Assessment of Body Composition

The Penn cohort underwent whole‐body DXA assessment using a Hologic densitometer (Delphi/Discovery Systems/Horizon Systems, *Hologic Inc., Bedford, MA*). The in vitro coefficient of variation for Hologic measurement of lean mass was less than 0.6% and the in vivo coefficient of variation in adults was less than 1% [12]. For the UCSF subjects, a Lunar Prodigy DXA system (software version 9.3) was used. The ESCAPE‐RA study also utilized a Lunar Prodigy DXA system (Prodigy software, version 05.60.003) (*GE/Lunar Radiation, Madison, WI*). In vivo coefficients of variation for measurement of lean mass by the Lunar Prodigy have been estimated at 1% or less [13]. Body composition measures for the UCSF subjects and ESCAPE RA subjects were adjusted based on the method by Shepherd et al. to facilitate comparison to data generated on Hologic equipment [14]. We utilized a previously validated method to quantify visceral adipose tissue area (VAT) area in the Penn cohort [15, 16].

### 2.3 Measurement of Circulating Adipokines

In the Penn and UCSF cohorts, adiponectin was measured on stored serum samples using a commercially available enzyme linked immunosorbent assay (ELISA) from R&D Systems. Leptin was measured using an immunoassay from *Meso Scale Discovery*. In the ESCAPE‐RA study, adiponectin and leptin were measured by ELISA at the Laboratory for Clinical Biochemistry Research (*University of Vermont, Burlington, Vermont, USA*). In order to facilitate comparison to the Penn and UCSF cohorts, 10 samples from ESCAPE‐RA were also assessed using the R&D Systems and *Meso Scale Discovery* assays.

### 2.4 Statistical Analysis

Appendicular lean mass index excluding bone content (ALMI, kg/m^2^) and fat mass index (FMI, kg/m^2^) were determined by dividing the respective estimate by height‐squared, similar to the calculation of body mass index (BMI). Measures of change for lean mass parameters were evaluated as continuous measures as well as dichotomized at 0 (any loss of lean v. gain or stable lean).

We aimed to evaluate whether loss of lean mass was associated with changes in adiposity over the same time period. Mixed effects linear regression models were established with the dependent variable defined as the change in FMI (in kg/m^2^) from baseline to follow‐up and the independent variable defined as a loss of ALMI (change in ALMI < 0 kg/m^2^) over the same follow‐up. Mixed effects logistic regression also evaluated the association between a loss of ALMI and a loss of FMI (where the outcome was whether the absolute change in FMI was ≥ or < 0). Models were adjusted for age at baseline, sex, race, baseline ALMI and FMI and study with independent correlation structure and cluster‐robust standard errors applied to account for clustering by study. We evaluated changes in visceral fat area and changes in leptin and adiponectin levels as secondary outcomes. The analysis also explored whether loss of ALMI was associated with greater loss of FMI *among those with greater FMI at baseline*. Analyses were also performed separately within each individual study and across gender to assess whether associations were distinct in the individual cohorts or among men and women. We also explored whether high levels of inflammation (high CRP) and the use of glucocorticoids modified key associations and contributed to change in adiposity over time.

## 3 Results

The detailed characteristics of the entire study cohort are summarized in Table 1. Of the 451 participants, 361 (80%) had a follow‐up visit at an average follow‐up time of 2.65 (0.71) years. Patients missing the follow‐up visit were older, more likely to be male and had lower ALMI at baseline (Table S1). On average, there was a small decrease in ALMI [Mean (SD): −0.021 (0.0.52)] and increase in FMI [Mean (SD): +0.12 (1.58)] during follow‐up (all *p* > 0.16). Out of 361 participants, 195 lost lean mass, while 166 gained or had a stable lean mass.

**TABLE 1:** Characteristics of patients included in the study, stratified by cohort.

	Combined	UCSF	Penn	ESCAPE RA
*N*	451	141	113	197
Follow‐up, *N*	361	120	83	158
Follow‐up time (yrs)	2.7 (0.71)	2	3.1 (1.1)	3
Age, (yrs)	58.3 (10.5)	58.6 (10.8)	54.2 (13.2)	59.4 (8.7)
Female %, (*N*)	58% (258)	60% (85)	54% (46)	60% (118)
Black, % (*N*)	14% (61)	4% (6)	34% (29)	10% (18)
Body mass index (kg/m2)	28.1 (5.9)	27.1 (6.0)	28.1 (6.8)	28.4 (5.3)
ALMI (kg/m^2^)	6.96 (1.41)	6.64 (1.18)	7.19 (1.40)	6.65 (1.34)
FMI (kg/m^2^)	10.67 (4.40)	10.55 (4.29)	10.69 (5.20)	10.74 (3.99)
Disease duration (yrs)	12 (5, 22)	18 (11, 27)	8 (3, 17)	9 (4, 17)
Current Smoking, % (*N*)	13% (57)	6% (8)	24% (20)	12% (23)
ACPA Positive, % (*N*)	72% (181/249)	66% (92)	82% (89)	—
CRP (mg/dL)	0.46 (0.13, 0.90)	0.19 (0.07, 0.52)	0.8 (0.5, 1.4)	0.27 (0.11, 0.78)
Pain (0–100)	23 (10, 48)	20 (10, 40)	40 (15, 65)	21 (8, 39)
Patient Global (0–100)	62 (36, 84)	—	35 (15, 50)	78 (56, 93)
Glucocorticoid use, % (*N*)	37% (155)	32% (43)	44% (49)	39% (74)
HAQ Score	0.85 (0.69)	0.94 (0.67)	0.79 (0.63)	0.82 (0.74)
Δ ALMI	−0.021 (0.52)	−0.059 (0.61)	0.10 (0.50)	−0.053 (0.62)
Δ FMI	0.12 (1.59)	0.080 (1.23)	−0.28 (1.90)	0.38 (1.61)

Abbreviations: ACPA = Anti‐citrullinated protein antibody; ALMI = appendcular lean mass index; BMI = body mass index; CRP = C‐reactive protein; CSA = cross‐sectional area; FMI = fat mass index; HAQ = health assessment questionnaire.

### 3.1 Primary Analysis

**FIGURE 1:**
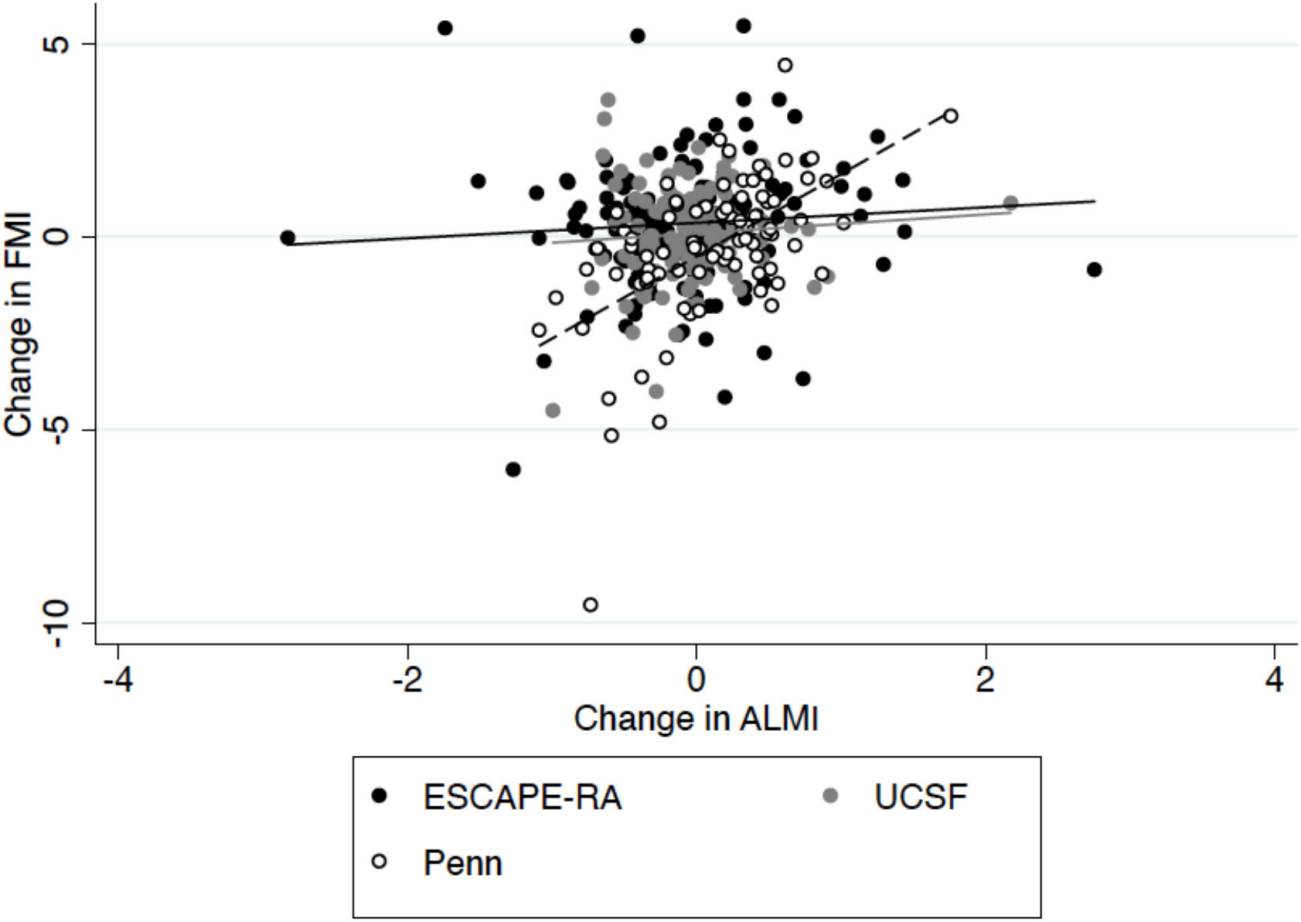
Unadjusted association between the change in FMI and the change in ALMI over time, stratified by the ESCAPE‐RA, USCF and Penn cohorts. Abbreviations: FMI = Fat Mass Index; ALMI = Appendicular Lean Mass Index; ESCAPE‐RA = Evaluation of Subclinical Cardiovascular Disease and Predictors of Events in RA Study; UCSF = University of California San Francisco.

In unadjusted analyses, there was a positive correlation between the change in the ALMI and the change in FMI (*N* = 361; Spearman Rho: 0.18, *p* = 0.0005); however, this was not statistically significant in mixed effects models (*N* = 361; *β*: 0.60 (95% CI: −0.37, 1.57) *p* = 0.22) (Figure 1). In adjusted models, those that lost ALMI (change in ALMI < 0) also lost an average of 0.17 kg/m^2^ of FMI, while those with an increase or stable lean mass had an average of a 0.46 kg/m^2^ increase in FMI (*p* = 0.07) (Table 2). Those that lost ALMI also were more likely to have lost weight (*p* < 0.001) and to have lost FMI [OR: 2.30 (95% CI: 1.31, 4.05) *p* = 0.004] (Table S2).

**TABLE 2:** Adjusted mean (95% CI) change in measures of adiposity according to the change in ALMI as derived from mixed effects regression models.

COMBINED COHORT				
Fat Measures	*N*	Lost ALMI	Stable/Gained ALMI	*p*
Δ BMI	195/166	−0.77 (−1.21, −0.33)	1.07 (0.56, 1.59)	< 0.001
Δ FMI	195/166	−0.17 (−0.48, 0.14)	0.46 (0.08, 0.83)*	0.07
Δ Visceral Fat Area (Penn)	29/54	−18.7 (5.42)*	−3.44 (3.92)	0.03
Adipokines				
Δ Leptin (per SD)	138/164	−0.10 (−0.22, 0.022)	0.010 (−0.037, 0.24)	0.13
Δ Adiponectin (per SD)	136/156	−0.094 (−0.12, −0.070)	−0.15 (−0.17, −0.12)*	0.04

*Note:* Models adjusted for sex, study, baseline ALMI, baseline FMI and baseline outcome variable (for adipokines and visceral fat).

Abbreviations: ALMI = Appendicular lean mass index; FMI = fat mass index; SD = standard deviation.

* *p* < 0.05 compared to no change.

### 3.2 Exploratory Analyses

Leptin levels declined among those that experienced a loss of ALMI; though this was not statistically different from those that did not lose ALMI. There was no significant increase in adiponectin levels (Table 2) among those that lost ALMI; however, reductions in adiponectin were significantly greater among those that did not lose ALMI (*p* = 0.04). Within the Penn cohort, visceral fat also declined to a greater degree among those that had a loss in ALMI (Table 2).

When explored separately in regression models, higher CRP at baseline (per 1 mg/dL) was associated with a greater loss of FMI [*β*: −0.011 (−0.019, −0.0027) *p* = 0.009]. Glucocorticoid use at baseline was not associated with changes in FMI (*p* = 0.41). Baseline ALMI was not associated with changes in FMI.

In regression models, higher baseline FMI was associated with significantly greater decline in FMI [*β*: −0.12 (−0.17, −0.07) *p* < 0.001]. This association was greatest among those that lost ALMI [*β*: −0.17 (−0.22, −0.12) *p* < 0.001] (p for interaction = 0.007), suggesting that those with the highest FMI at baseline lost the greatest amount of FMI in the setting of muscle loss (Figure S1). There were no differences in the observed associations between men and women (*p* for interaction = 0.98). CRP levels and glucocorticoid use also did not modify associations between loss of muscle and loss of FMI.

A more negative change in FMI was observed among those that lost lean mass among all studies, though the effect was most pronounced in the Penn cohort [*β*: −1.84 (95% CI: −2.56, −1.11), *p* < 0.001] (Figure 1, Table S3).

## 4 Discussion

In this study of patients with established RA, those that lost lean mass during follow‐up were more likely to also lose fat mass, visceral fat and to have reductions in circulating leptin levels. The primary advance of this study is to suggest that the development of muscle loss in RA is likely to be associated with loss of fat stores. These longitudinal changes in body composition are not consistent with cross‐sectional patterns that suggest simultaneous loss of lean mass and gain of fat mass. Thus, these observations suggest a need to revisit the commonly cited paradigm. Between those that lost muscle compared to those that did not, the difference in the change in FMI was 0.63 kg/m^2^ on average, which represents a modest difference of about 1.8 kg or 4 pounds of fat for a human of average height. Those that lost ALMI also had more than twice the odds of losing fat.

Several prior cross‐sectional studies suggested that, on average, patients with RA have low lean mass as well as normal or excess fat mass [4, 5, 6, 17, 18]. For example, Engvall et al. found that 50% of patients with RA had lean mass below the 10th percentile for healthy adults while 45% had FMI above the 90th percentile [4]. Prior studies by Roubenoff also found that patients with RA had low estimates of muscle mass despite similar BMI compared to controls [5]. These observations have reasonably been interpreted to signal that the effect of RA inflammation leads to the simultaneous loss of lean mass and increase in fat mass, resulting in a normal BMI.

To reconcile the observations from this study conducted over time with prior cross‐sectional observations, one must consider alternative models. The observation in the current study that *muscle loss occurs in tandem with loss of fat mass* suggests that the development of sarcopenia and excess adiposity observed in RA do not generally occur simultaneously as part of the same process. Several alternative models are plausible. Firstly, patients with treated RA may gain adipose tissue with aging, perhaps during periods of relatively low disease activity, low physical activity and use of glucocorticoids. Patients that gain or regain weight may not have complete resolution of muscle deficits that have previously occurred or may gain proportionally less muscle during periods of weight gain (Figure S2). In support of this, a prior study in older adults suggested that muscle loss is proportionally greater during periods of weight loss than the proportional muscle gain during periods of weight gain [19]. Cross‐sectional observations may also stem from the result of muscle loss occurring in individuals that were obese at the time of their development of the disease. In this context, proportionally greater loss of lean mass compared to the rate of loss of adipose tissue could result in a combination of low lean mass and ongoing excess adipose tissue within an individual.

Redistribution of muscle and fat is not a phenomenon that is limited to patients with RA and is observed in normal aging and in other chronic conditions. High levels of inflammatory cytokines, including TNF, induce muscle atrophy through both direct effects as well as through growth hormone resistance [20] while also promoting lipolysis in adipose tissue through several mechanisms. Inflammation generated from excess adiposity may also contribute to this process, perhaps increasing the risks of muscle loss.

The observation that loss of muscle occurs in tandem with loss of weight and adiposity in RA is in line with other settings where sarcopenia and cachexia are often described, such as advanced cancer. Because sarcopenia often occurs because of an increased energy demand or low energy availability, catabolism of muscle is observed along with lipolysis or breakdown of stored fats to meet energy requirements [21]. Weight loss is a key criterion of cancer cachexia and weight is closely tracked in oncology clinics [22]. RA may be distinct from other entities in its chronicity and periods of low and high disease activity, which may result in weight cycling [23, 24], a phenomenon that may have metabolic consequences and implications for fat distribution over the long‐term.

Studies have also described redistribution of adipose tissue in patients with RA, with higher levels of visceral fat [25]. In this study, we found that those that lost muscle had significantly greater loss of visceral fat as well, suggesting that the visceral compartment is not an exception to the general observations from the study. The findings of the current study are consistent with those observed in a mouse model of rheumatoid cachexia in which a reduction in visceral fat was observed for mice with collagen induced arthritis compared to controls [26]. Overall, these findings suggest that most patients do not simultaneously lose muscle and redistribute fat to the visceral compartment. One must consider that accumulation of visceral adipose tissue may also occur through a distinct process from muscle loss in RA.

Among those that lost lean mass, loss of fat was more substantial among those with greater adiposity at baseline. This may reflect that those with greater adiposity simply have the greatest opportunity to lose it. However, inflammation related to excess adiposity may also promote muscle atrophy and lipolysis [20]. Thus, it is possible that local and systemic inflammation among those with excess adiposity at baseline might contribute directly to a greater risk of subsequent declines in both muscle and fat. In a prior study in a Korean general population of adults, greater visceral adiposity predicted a greater loss of muscle over time, an observation that is consistent with those of the current study [27]. Differences in adiposity at baseline may partly explain the differences in the strength of the association across the three cohorts, though this also might be related to other population characteristics or differences in DXA methods.

This study aimed to describe changes in fat mass occurring alongside muscle loss. This study design is unable to determine whether loss of fat *causes* loss of muscle or vice versa, but rather can only describe the observation that they tend to occur in tandem. While the use of three distinct study populations was a strength of the current study, the combination of three cohorts is also a challenge since the DXA manufacturer, assessments and available data varied by population. The combination of multiple cohorts, with distinct methodologies and populations, is likely to have added variability to the study that would be most likely to bias results toward the null. Further, the lack of a control group limits the ability to directly compare findings observed in this RA population to what might be expected with normal aging or with other conditions. Loss to follow‐up was related to a number of factors at baseline that suggest that sicker participants were less likely to attend a follow‐up visit. While difficult to predict, it therefore seems likely that we may have underestimated the amount of change occurring over time due to loss to follow‐up in sicker individuals.

The strengths of this study are the use of longitudinal data from three distinct cohorts and the availability of multiple approaches to assessing changes in adiposity including an evaluation of visceral adiposity and adipokines. The longitudinal aspects of the study are the primary advance over existing cross‐sectional studies.

In summary, in this longitudinal cohort, muscle loss in RA was more likely to occur in the context of loss of both total and visceral fat. This study supports the hypothesis that thin patients and those that have experienced periods of weight loss are the most likely to have experienced a reduction in lean mass, informing potential screening practices and interventional approaches for sarcopenia and cachexia.

## Funding

This work was supported by a Veterans Affairs Clinical Science Research and Development Merit Award (I01 CX001703) and a VA Clinical Science Research and Development Award (IK2 CX000955). This work was also supported by NIH grants K12 HD068373 (DRW), the University of Pennsylvania Clinical and Translational Research Center (UL1 RR024134), a NIAMS grant P60‐AR053308 and the National Center for Research Resources through a University of California San Francisco Clinical and Translational Sciences Institute grant UL1‐RR024131. The ESCAPE RA Study was funded by NIH NIAMS AR050026–01 (P.I. Joan Bathon). Additional support was provided by the Johns Hopkins Bayview Medical Center General Clinical Research Center (Grant Number M01RR02719). KDW is supported by a VA Career Development Award (IK2CX002351).

## Conflicts of Interest

JFB has received consulting fees from CorEvitas LLC, Formation Bio and Burns‐White LLC.

## Supporting information


**Table S1:** Characteristics of study participants who did not complete a follow‐up visit v. those who did not complete a follow‐up visit.
**Table S2:** Odds ratio for losing FMI Z‐Score or FMI (kg/m2) among those that lost ALMI Z‐Score or ALMI (kg/m2), respectively, stratified by cohort.
**Table S3:** Association between loss of lean mass (ALMI) and change in fat measures stratified by study. The estimate represented is the beta‐coefficient from regression models.
**Figure S1:** Association between the change in FMI with the baseline FMI among those that lost ALMI during follow‐up. The greatest change in FMI Z‐Score is observed among those with the greatest FMI at baseline.
**Figure S2:** Conceptual model explaining why both expansion of body fat and reduction in muscle mass may occur in RA, due to periods of weight cycling.
