# Supplementary material for: Loss of Lean Mass in Rheumatoid Arthritis Is Associated With Loss of Total and Visceral Fat
Source: J Cachexia Sarcopenia Muscle. 2026 Feb 11;17(1):e70229. doi: 10.1002/jcsm.70229 (PMC12894774; doi:10.1002/jcsm.70229)
Supplement: Supplementary file 1 — Table S1: Characteristics of study participants who did not complete a follow‐up visit v. those who did not complete a follow‐up visit. Table S2: Odds ratio for losing FMI Z‐Score or FMI (kg/m2) among those that lost ALMI Z‐Score or ALMI (kg/m2), respectively, stratified by cohort. Table S3: Association between loss of lean mass (ALMI) and change in fat measures stratified by study. The estimate represented is the beta‐coefficient from regression models. Figure S1: Association between the change in FMI with the baseline FMI among those that lost ALMI during follow‐up. The greatest change in FMI Z‐Score is observed among those with the greatest FMI at baseline. Figure S2: Conceptual model explaining why both expansion of body fat and reduction in muscle mass may occur in RA, due to periods of weight cycling. [file JCSM-17-e70229-s001.docx]

**Supplementary Material**

Supplementary Table S1. Characteristics of study participants who did not complete a follow-up visit v. those who did not complete a follow-up visit.

|  | Not Missing Follow-up | Missing Follow-up Visit | p |
| --- | --- | --- | --- |
| Age, (yrs) | 57.8 (10.2) | 60.6 (11.5) | 0.02 |
| Female %, (N) | 244 (69%) | 47 (52%) | 0.002 |
| Race  Black, % (N) | 44 (12.5%) | 18 (19.8%) | 0.07 |
| Study |  |  |  |
| ESCAPE-RA | 151 (43%) | 39 (43%) | 0.05 |
| UCSF | 120 (34%) | 21 (23%) |  |
| Penn | 83 (23%) | 30 (34%) |  |
| BMI (kg/m2) | 28.3 (6.1) | 27.7 (6.3) | 0.40 |
| ALMI Z-Score | -0.32 (0.99) | -0.56 (1.06) | 0.05 |
| FMI Z-Score | -0.22 (1.07) | -0.31 (1.23) | 0.50 |
| ALMI_FMI_ Z-Score | -0.27 (1.33) | -0.51 (1.38) | 0.13 |
| Disease duration (yrs) | 13.8 (11.0) | 17.0 (12.5) | 0.02 |
| Current Smoking, % (N) | 40 (11.3%) | 17 (18.9%) | 0.06 |
| HAQ | 0.83 (0.69) | 0.96 (0.75) | 0.12 |

Abbreviations: ESCAPE-RA= Evaluation of Subclinical Cardiovascular Disease and Predictors of Events in Rheumatoid Arthritis; UCSF= University of California San Francisco; BMI= Body Mass Index; ALMI= Appendicular Lean Mass Index; FMI= Fat Mass Index; ALMI_FMI_= Adiposity-adjusted Appendicular Lean Mass Index; HAQ= Health Assessment Questionnaire

Supplementary Table S2: Odds ratio for losing FMI Z-Score or FMI (kg/m^2^) among those that lost ALMI Z-Score or ALMI (kg/m^2^), respectively, stratified by cohort.

| **Loss of FMI (kg/m^2^)** | **N** | **OR (95% CI)** |  |
| --- | --- | --- | --- |
| ESCAPE-RA | 158 | 2.15 (1.03, 4.48) | 0.04 |
| UCSF | 120 | 1.64 (0.72, 3.76) | 0.24 |
| Penn | 83 | 6.14 (2.04, 18.5) | 0.001 |
| Combined | 361 | 2.30 (1.31, 4.05) | <0.001 |

Supplementary Table S3: Association between loss of lean mass (ALMI) and change in fat measures stratified by study. The estimate represented is the beta-coefficient from regression models.

| **A.** | **UCSF (N=120)** | **Penn (N=83)** | **ESCAPE-RA (N=151)** |
| --- | --- | --- | --- |
|  | **Lost ALMI v. Gained ALMI** | **Lost ALMI v. Gained ALMI** | **Lost ALMI v. Gained ALMI** |
| **Fat Measures** | *β (95% CI)* | *β (95% CI)* | *β (95% CI)* |
| Δ FMI (group difference) | -0.19 (-0.64, 0.25) | -1.84 (-2.57, -1.11) | -0.37 (-0.90, 0.15) |
| **Adipokines** |  |  |  |
| Δ Leptin (per SD) | 0.11 (-0.15, 0.37) | -0.55 (-0.84, -0.25) | -0.14 (-0.30, 0.01) |
| Δ Adiponectin (per SD) | 0.064 (-0.12, 0.25) | 0.11 (-0.20, 0.41) | 0.15 (-0.04, 0.33) |

Models adjusted for age, sex, race, study, baseline ALMI Z-Score, baseline FMI Z-Score,

Supplementary Figure S1. Association between the change in FMI with the baseline FMI among those that lost ALMI during follow-up. The greatest change in FMI Z-Score is observed among those with the greatest FMI at baseline.

Supplementary Figure S2: Conceptual model explaining why both expansion of body fat and reduction in muscle mass may occur in RA, due to periods of weight cycling.
